# Modeling and simulation of biological systems from image data

**DOI:** 10.1002/bies.201200051

**Published:** 2013-03-27

**Authors:** Ivo F Sbalzarini

**Affiliations:** MOSAIC Group, Max Planck Institute of Molecular Cell Biology and GeneticsDresden, Germany

**Keywords:** computational biology, image-based systems biology, simulation, spatiotemporal modeling, systems biology

## Abstract

This essay provides an introduction to the terminology, concepts, methods, and challenges of image-based modeling in biology. Image-based modeling and simulation aims at using systematic, quantitative image data to build predictive models of biological systems that can be simulated with a computer. This allows one to disentangle molecular mechanisms from effects of shape and geometry. Questions like “what is the functional role of shape” or “how are biological shapes generated and regulated” can be addressed in the framework of image-based systems biology. The combination of image quantification, model building, and computer simulation is illustrated here using the example of diffusion in the endoplasmic reticulum.

## Introduction

Since the term “Systems Biology” 1, 2 has been coined, it has been used to designate a number of different things. This ranges from biological applications of systems theory 3 to automated high-throughput experiments followed by statistical data mining 4 to computational modeling and simulation of biological processes 5, 6. The common theme that seems to emerge in systems biology is a focus on dynamics and interactions, which are believed to cause the apparent “complexity” of life. Often, the goal is to formulate predictive models of these interactions from systematically collected quantitative data 7. This approach is rooted in the mechanistic philosophy that if we can predict the behavior of a system from its current state (the data) and first principles of chemistry and physics, then we have understood how the system works 8.

Many biological phenomena of interest, such as the intracellular localization of molecules, morphogenesis, growth, and forest distribution dynamics involve a spatial component that calls for a spatiotemporal systems understanding. Accounting for the spatial localization and distribution of a system's constituents immediately brings into play the shapes and geometries of things, as well as their deformations over time. How are biological shapes generated and controlled? What is the functional role of shape? How do cells organize into tissues? Why is the endoplasmic reticulum (ER) a network of tubules and lamella, rather than a single spherical compartment 9? These are fundamental questions involving temporal dynamics of spatial distributions. In image-based systems biology, shapes, spatial distributions, and their temporal dynamics are extracted from images. This interprets images as *quantitative measurements*, rather than mere visualizations, and renders them a primary data source for systems biology, complementing various -omics data.

Using images as quantitative measurements, however, raises a couple of key issues. First, we need to be able to reproducibly extract quantitative information from images. Second, we must know the accuracy (error bars or confidence intervals) of the extracted information in order to decide whether a certain conclusion is supported by the data or could just be an artifact of measurement errors 10. Third, we have to express and account for prior knowledge and hypotheses about the system we study. Fourth, we need versatile methods that can be applied in more than just one specific situation. Fifth, we need user-friendly and efficient software that facilitates transfer of new methods into daily scientific practice.

This essay gives an introduction to the concepts, challenges, methods, and terminology of image-based systems biology. The emphasis is on exposing general concepts and unifying ideas. I follow the typical workflow of image-based systems biology: from image analysis to model building and simulation (“in silico experiment”). I focus on *computational* methods for image quantification and simulation. This may not always be the best choice, as manual or theoretical methods are preferable in some cases. Thus, I first provide a few indications of why and when computational methods can be useful or even indispensable.

**Figure 1:**
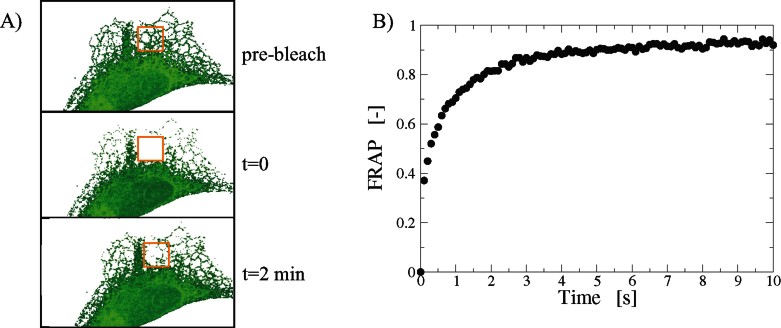
Example of a FRAP experiment with ssGFP-KDEL (pure GFP with an ER targeting and retention sequence) expressed in a VERO cell (data: Helenius lab, ETH Zurich). **A:** A time-lapse sequence of confocal micrographs before bleaching (top), immediately after bleaching the region of interest (ROI) given by the orange square (middle), and 2 minutes after bleaching (bottom). For each time point we measure the total fluorescence intensity in the ROI, relative to the pre-bleach intensity. **B:** FRAP curve showing the fluorescence recovery due to influx of unbleached protein into the bleached region. This influx only happens along ER tubules and hence depends on the geometry of the organelle in the vicinity of the ROI.

**Figure 2:**
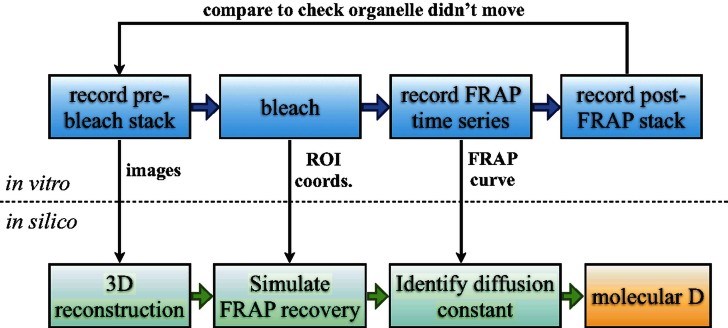
Workflow of the example used throughout this text. We consider the problem of using fluorescence recovery after photobleaching (FRAP) experiments 13, 14 to measure the molecular diffusion constant in a complex-shaped organelle, the endoplasmic reticulum (ER) 11, 12. The workflow of the image-based solution starts from recording a pre-bleach confocal *z*-stack, which is used to reconstruct the ER geometry in 3D in the computer. This reconstruction is then used for in-silico simulation of the FRAP recovery dynamics in the same geometry and with the bleached region of interest (ROI) at the same location as in the experiment. Comparing the simulation output with the experimentally measured FRAP curve then allows the identification of the unknown molecular diffusion constant *D* of the fluorescently tagged protein. Finally, a post-FRAP *z*-stack is recorded to check that the organelle has not significantly moved or deformed during the course of the experiment.

In order to illustrate how the different bits and pieces form a coherent workflow, I carry through the entire essay an old example from my own work: studying the influence of organelle geometry on diffusion processes in the ER 11, 12, as observed in fluorescence recovery after photobleaching (FRAP) experiments 13, 14 (see Fig. 1). This work addressed a twofold goal: on the one hand, we wanted to have a quantitative tool to measure molecular diffusion constants in complex-shaped organelles, on the other hand, we wanted to study the effects of organelle shape on transport processes. The first goal requires modeling because the diffusion constant is not directly observable in a FRAP measurement, since the fluorescence recovery dynamics measured by FRAP also depend on the geometry of the organelle. If more or thicker ER tubules lead into the bleached region, recovery is faster for identical diffusion constants. The second goal requires modeling because the diffusion constant is not controllable in the experiment; we cannot dictate to the cell what diffusion constant a protein should have. While we can observe FRAP dynamics in differently shaped ERs, we are never sure whether the observed differences in recovery dynamics come from geometric differences or from differences in the molecular diffusion constants in the different cells. In a computer simulation, however, we can fix the diffusion constant to any value we like and hence separate its effect from the effect of geometry. In this example, we only consider observations on length scales larger than individual ER tubules and on the time scale of seconds. Other experimental techniques to measure diffusion constants, such as fluorescence correlation spectroscopy 15 or single-molecule tracking 16, 17, can be used as independent validations, but the present model does not reproduce the single-molecule dynamics they measure. The workflow and data flow of this example is summarized in Fig. 2. This is a simple example of image-based systems biology, where quantitative imaging is used to build a predictive model that enables learning a non-observable quantity.

## Whither computers?

The aim is to model and simulate spatiotemporal dynamics and interactions in the real shape context of the biological system. The shapes, their dynamics, and the spatiotemporal distributions of the players (e.g. fluorescently labeled proteins) are quantified from images. This mainly requires four iterative steps: (i) image analysis and image quantification, (ii) model formulation, (iii) simulations of the model, and (iv) model validation and parameter identification. Each of these steps can be done either manually, theoretically (i.e. with paper and pencil), or computationally. Computer simulations do not *solve* a model, but only punctually probe its behavior for specific parameter values (e.g. diffusion constants and reaction rates) and at specific locations in space (called “discretization points”). Computer simulations are thus more akin to experiments than to theory, which is why they are sometimes referred to as “in silico experiments”.

The following properties of biological systems may hamper their theoretical treatment 18. Biological systems tend to be:

- *Hierarchically organized*: We think of biological systems as organized in levels where the smaller constitutes the larger 19. Atoms constitute molecules that constitute organelles that constitute cells that constitute tissues that constitute organs that constitute organisms that constitute ecosystems. This is akin to how computer software is organized, where characters constitute keywords that constitute code lines that constitute objects or subroutines that constitute programs that constitute software systems. Hierarchy is well expressed in the framework of computation 20, computational complexity 21, and algorithms 22.
- *Coupled across scales*: In biology, events on one scale can influence dynamics at any other scale, not only at the immediately adjacent ones. Examples include quorum sensing in bacteria 23 and the behavioral changes in animals upon binding of neurotransmitters or hormones to their receptors. Capturing these cross-scale effects requires multi-scale modeling techniques 24. In many passive (dead) systems, such scale-coupling does not exist, allowing models to be formulated at a certain level of description without requiring information from other levels. Mechanics, for example, can describe the bending of a rod under load without needing information about the positions and velocities of the individual atoms that constitute the rod. Such scale separation is frequently not obvious in biology.
- *Regulated*: Most biological systems possess sensors with which they constantly monitor their state and actuators to react to perturbations. It is easy to predict from the laws of physics what trajectory a tennis ball will follow when thrown. It is, however, virtually impossible to predict the trajectory of a thrown cat. This is because the cat constantly monitors its flight and uses limbs and tail to steer, making sure it lands on its feet.
- *Complex-shaped*: With the exception of a few unicellular organisms, living things have complex and irregular shapes that moreover grow, move, and deform over time 25. These shapes are not only difficult to describe mathematically, but they can also qualitatively alter the dynamics of processes within 11, 26. Moreover, model equations are often impossible to solve theoretically in complex domains.
- *Plastic*: The dynamics of a biological system change over time. Examples include cell-cycle-dependent transcription, the change of physiological dynamics with age, and immune reactions to pathogens. We thus need to deal with models that change their parameters or even their structure over time. It is often hard to intuitively understand indirect feedback via model changes, but computer simulations may help disentangle the different influences.
- *Non-equlibrium*: While living systems can be at steady state, they are never at equilibrium 27. Much of physics has been developed for equilibrium situations and does not immediately transfer to biology. The kinetics of biochemical reactions in live cells and small volumes, such as organelles, for example, is markedly different from equilibrium kinetics as governed by the macroscopic law of mass action 28–33. Since our theoretical knowledge of non-equilibrium dynamics is incomplete, computer simulations are often the best resort.
- *Nonlinear*: Bimolecular reactions, cooperation, feedback loops, and competition are important concepts in biology. These and others render the system dynamics nonlinear. Many nonlinear models are impossible to solve theoretically and our intuition of how a nonlinear system reacts to perturbations is often wrong 34, because a nonlinear system is not equal to the sum of its parts. Again, computer simulations are often the best way out.

Due to these properties, biological systems are often called “complex”. This, however, has little to do with the mathematical concept of complexity as used in computer science 21, but often rather means that we do not fully understand them. Given these properties of biological systems, the use of computational data analysis and simulation is indicated whenever:

1. the amount of data is too large for manual analysis,
2. reproducibility of the analysis is important,
3. the system dynamics cannot be intuitively understood,
4. Time or length scales are outside of the experimentally accessible range,
5. quantities of interest are not experimentally observable or controllable, or
6. ethical considerations make experiments undesirable.

## The first step: Image analysis and quantification

Image-based systems biology combines systematic quantitative image data collection with spatiotemporal systems modeling. This naturally starts from imaging, followed by detecting, delineating, and reconstructing the shapes and concentration distributions of interest from the images. In addition to many challenges in sample preparation, labeling, and image acquisition not discussed here, computational bio-image analysis comes with its own set of difficulties.

These are best understood by considering the nature of an image as a measurement of a real-world scene, for example, the spatial distribution of a fluorescent marker. There are many ways a scene or sample can be imaged: using different imaging modalities, different microscopes, different magnifications, different view angles, etc. A specific *view* leads to one of all possible images. Clearly, information is lost from the real specimen, as only one or few of the many possible views are recorded. The optics of the microscope then map the view to an intensity distribution in the focal plane. This entails a further loss of information, as no microscope has a perfect point-spread function (PSF) 35. Light diffraction leads to a PSF of non-zero width, preventing the separation of objects close together. The minimum gap required between two objects such that they are seen as separate in the image is called the *resolution* of the microscope; it is comparable to the wavelength of the recorded light, with different microscopy techniques having different pre-factors. In addition, nonlinear effects such as aberrations occur, even in aberration-corrected lenses. The resulting blurry intensity distribution in the focal plane is then discretized onto the pixel grid of the camera sensor, where each pixel measures the total intensity in its region of the focal plane. This measurement, however, is subject to various sources of noise and measurement errors. For example, in fluorescent imaging the signal is often dim with a small number of photons collected from the specimen in each pixel, causing Poisson noise. Other types of noise such as Gaussian noise are also introduced from the electronics used to detect low levels of photons. In the end we thus observe a noisy, discretized, blurred map of the real radiance distribution in the imaged sample.

A digital image is a table of numbers, where each entry is the recorded intensity in a given pixel. Imagine you are given such a table, rather than its visualization as an image, and you are asked to find and delineate objects represented in the image. All you are allowed to do is apply arithmetic operations to the numbers in the table. In the end, this should result in a new table containing the number, sizes, positions, shapes, etc. of the objects represented in the image, e.g. the cell nuclei in the imaged tissue. The challenge when designing image-analysis methods is to find (and program into a computer) a sequence of arithmetic operations that reliably does this job for previously unseen input images. Extracting from an image the positions, shapes, and brightnesses of all fluorescently labeled nuclei reduces the amount of data from one number per pixel to a few numbers per nucleus; the table of nuclei is smaller than the original table of pixels. It does, however, increase the utility of the information, as we can biologically reason about nuclei, but not about pixels.

Another big challenge in bio-image analysis is to quantify the errors and uncertainties in the results. Images are often under-used with only a fraction of the information contained in them actually extracted and studied. Without uncertainty quantification 36, 37, however, we will never know whether an observed variation in the read-out comes from imaging noise, image-analysis errors, or real biological differences in the samples. Approaches that address these issues exist 38, but much research remains to be done in this direction.

**Figure 3:**
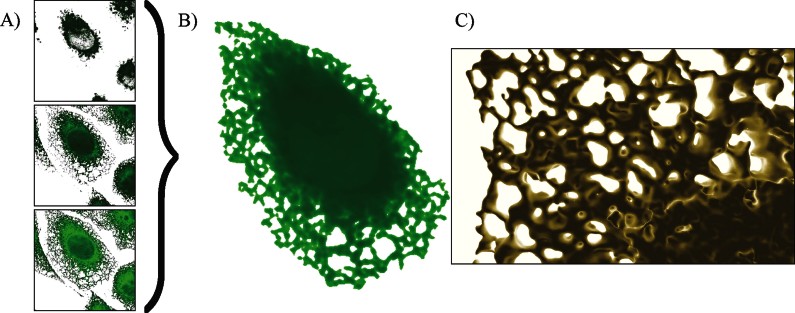
Image processing in the ER FRAP example 11. **A:** Three example slices from a confocal pre-bleach *z*-stack of the fluorescently labeled (ssGFP-KDEL) ER in a VERO cell (images: Helenius lab, ETH Zurich). **B:** Using per-pixel thresholding, the 3D shape of the ER is reconstructed in the computer as an intensity iso-surface. **C:** Magnification of a part of the geometry to illustrate the level of detail of the reconstruction.

In our ER example 11 we used the simplest possible approach to determining which pixels are inside the ER and which are outside. While this turned out to be sufficient for the given application, we note that much more sophisticated image-analysis methods and software are available when needed 39–43. In our example it was sufficient to fix a threshold intensity value and instruct the computer to go through the table of pixel intensities from the entire 3D *z*-stack and flag all pixels with an intensity above the threshold as “ER”, all others as “background”. The membrane of the ER is then the surface enclosing all “ER” pixels or, equivalently, the intensity iso-surface at the threshold value, as shown in Fig. 3.

## The second step: Modeling

Once shapes, objects, intensities, or motion trajectories 17 are quantified from images, this information can be used to formulate models of the system under study. The domain of computational models (as opposed to model organisms, thought models, cardboard models, or others) comprises four kingdoms: discrete/stochastic, discrete/deterministic, continuous/stochastic, and continuous/deterministic models. Each of these kingdoms contains a wealth of phyla, classes, and families. The kingdom largely determines how a model has to be simulated and what can be expressed with it.

The dynamics of a deterministic model is completely determined by the model's present state. In contrast, the evolution of a stochastic model involves a random component and future states are not predictable. The only thing one can predict from a stochastic model is the probability distribution (viz., the mean, variance, etc.) of the future states. This is much like the weather forecast using a computational model of atmospheric physics in order to tell you that that there is a 65% chance for rain. Only a probability can be given, because there are too many random or unknown influences onto the weather. Continuous models describe the evolution of variables that take continuous values, such as the real-valued concentration of a chemical. In discrete models, the variables are constrained to assuming only integer multiples of a certain unit. An example is the number of molecules in a compartment. This number can be 1, 2, 3, …, but it cannot be 1.5, hence defining a discrete variable over the unit “molecule”.

We distinguish mechanistic (“bottom-up”) from phenomenological (“top-down”) models. Mechanistic models reproduce emerging behavior from the next finer level of description, whereas phenomenological models do not. An example of the former is the prediction of the shape of the inner mitochondrial membrane, as observed in electron tomography images, from a continuous/deterministic model of lipid membrane mechanics under tension 44. Examples of the latter include reaction-diffusion models that reproduce various animal fur coats and seashell patterns 45–47. Moreover, we distinguish quantitative models from qualitative models. Quantitative models predict or reproduce the numerical values of the observables of interest, e.g. the concentration in nM of the output species of a signal transduction network. Qualitative models do not reproduce absolute levels or values, but only their relative changes. A qualitative model of the same signal transduction network would hence not tell us the output concentration, but only whether it goes up or down, or whether it is greater or less than a threshold level.

It is essential that any model strike a balance between level of detail and simplicity 48. Ockham's razor 49 states that every system should be modeled only with as much detail as necessary, and with as little as possible. The simplest model is the most useful one, the one from which we can learn the most about the key mechanisms at work. Much of the art of modeling consists in identifying the appropriate level of detail. Validation then shows whether neglected details are indeed insignificant for the process under study (see Verification and Validation Section).

In the ER FRAP model 11, we opted for a continuous/deterministic description. This was motivated by three reasons: First, the fluorescent markers are abundant in the ER, so we can consider the fate (position and velocity) of individual molecules negligible. Second, the diameter of even the thinnest ER tubule is orders of magnitude larger than a single protein. Third, the time scale we are interested in is the diffusive recovery time, which is several seconds. Individual thermal fluctuations of the molecules and bond vibrations are much faster than that and we do not need to resolve them. We can thus model the spatiotemporal evolution of a continuous fluorescence concentration field, which is also what we observe in the images as a spatial intensity distribution. Furthermore, we assume normal, homogeneous, and isotropic diffusion 11. We would only invoke more elaborate transport models if this simplest model failed to explain the data (Ockham's razor 49). Indeed, we found that normal, isotropic diffusion explains the data well, and that apparent anomalies in FRAP dynamics 50 can be explained by geometric effects from the shape of the organelle 11.

## The third step: Simulation

A number of software packages are available to simulate spatiotemporal models in biology 51–55. For many applications, however, it is still necessary to implement custom-made simulation programs that account for specific needs or include more recent simulation methods that are not yet available in standard software packages. It is important not to confuse the software tool with the method it implements. Most software implements more than one method, and most methods are implemented in various software tools. Depending on the kingdom of a model, different simulation methods are available to probe the model's behavior in silico 48.

Simulation methods for discrete/stochastic models represent the discrete objects (molecules, animals, cells, etc.) as individual numbers or objects in computer memory, whose interactions are governed by random numbers. In methods for discrete/deterministic models, discrete representations of objects interact according to deterministic rules. This means that the result of an interaction only depends on the positions and properties of the interacting entities; there is no random component. Continuous/stochastic models involve continuous distributions, or fields, that are defined everywhere in space and evolve according to a random process. Since a computer can only store a finite amount of numbers, but fields are defined at an infinite number of points in space, the fields need to be *discretized* before they can be simulated. Discretization involves selecting a finite set of *discretization points* where the field values are stored and their evolution is simulated over time. The discretization points play the role of weather stations. Only at the locations of the weather stations the air temperature is recorded and its evolution tracked over time. Since this delivers no information about the (temperature) field between individual discretization points, these points need to be sufficiently close together so we do not miss any interesting variations between them. The distance between neighboring discretization points is called the *resolution* of the simulation. In simulations of continuous/deterministic models, the continuous fields are discretized as described above. Their evolution, however, follows deterministic rules, such that the change of a field only depends on the fields at present.

**Figure 4:**
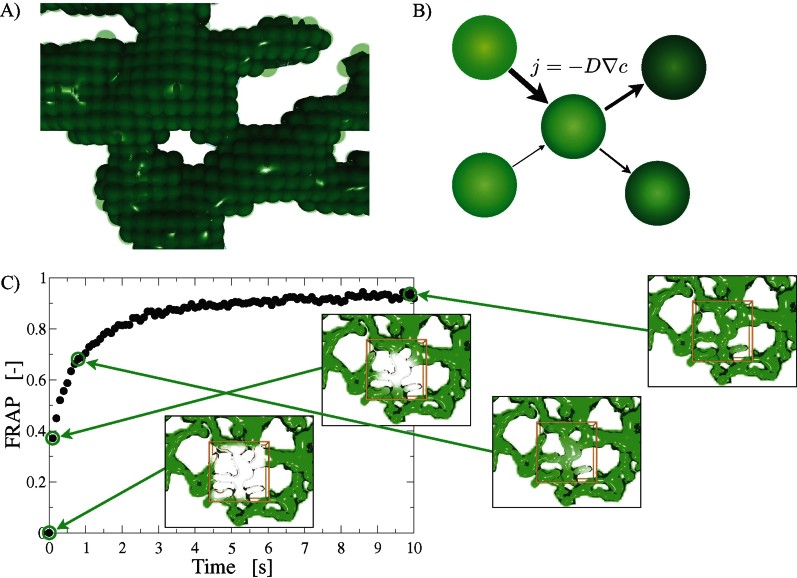
Simulation of a continuous/deterministic diffusion model in image-derived ER geometries 11. **A:** The ER is “filled with particles” that discretize the fluorescence concentration field. Each particle contains a certain amount of fluorescence. Particles in the bleached region are initially empty (not shown). **B:** In order to simulate the process of diffusion, particles exchange fluorescence with their neighbors according to Fick's law, which states that the flux *j* between any pair of particles is given by the concentration gradient *▿c* between these two particles, multiplied with the diffusion constant *D*. In each time step of the simulation, all particles interact with their neighbors according to this deterministic rule 56. In the figure, bright particles contain more fluorescent protein; the magnitudes of the fluxes are reflected by the thicknesses of the arrows. **C:** As the simulation steps forward through time, the FRAP curve can be computed by summing up the total fluorescence of the particles in the bleached region (orange box) at each time point. This leads to a simulated FRAP curve and allows visualizing the 3D intensity distribution over time (insets).

The continuous/deterministic ER FRAP model 11 considered as an example in this essay has been simulated using the method of particle strength exchange (PSE) 56, as illustrated in Fig. 4.

### Geometry representation in the computer

A challenge in any simulation method for image-based systems biology is the need to represent the image-derived geometries in the computer and to simulate the model in these often irregular geometries. The geometries can be arbitrarily complicated and may move and deform over time. Since a computer can only store numbers, shapes also need to be represented numerically. This can be done using a variety of methods 57, including triangulated surfaces 58, pixel/voxel sets 59, and implicit surface representations such as level sets 60 or phase fields 61. Level-set methods are well suited for simulating moving and deforming geometries 6, 62, 63.

Simulating a model on a curved surface is more difficult than simulating the same model in a volume. This is because the curvature of the surface needs to be explicitly accounted for, which often requires that it first be computed or numerically approximated. If the surface additionally moves or deforms over time, possibly in function of the local concentration of the substances diffusing on it, the problem gets even more complicated 62, 63. In the ER FRAP example 11, the geometry of the ER was represented as a level set 64, and the method has also been extended to simulating diffusion in the ER membrane 12.

## The fourth step: Parameter identification and model validation

Most models contain parameters such as rate constants, diffusion constants, compartment volumes, etc. If the values of all parameters are known or have been measured beforehand, the model is called *white*-*box* and can directly be simulated without any further ado 65. Models with unknown parameter values are called *black-box*. Estimating or inferring the unknown values of model parameters from data is called *parameter identification*; it is a well-researched topic in systems theory 66–68 and often the primary purpose of a model.

### Parameter identification

**Figure 5:**
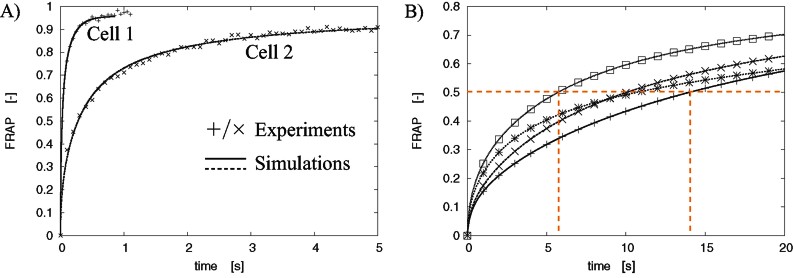
Parameter identification in the ER FRAP example 11. **A:** The unknown molecular diffusion constant is the only parameter in the model. It can be identified by fitting simulation output to experimental FRAP measurements, as shown. The dynamics in different cells is markedly different due to different ER geometries. Nevertheless, the diffusion constants that lead to the best fit are in the same range of 34 ± 0.95 µm^2^/seconds for ssGFP-KDEL in the ER lumen of VERO cells. This shows that a large portion of the observed variability in FRAP curves could be due to geometric effects. **B:** Quantification of the non-controllable geometric effects using the in silico model. Using the same molecular diffusion constant in simulations in different reconstructed ER geometries causes the recovery half-time (orange dashed lines) to vary by about 250%. This variation is purely geometry-induced. The fact that we can control the diffusion constant in the simulations allows disentangling the effects of geometry from the effects of molecular diffusion.

In order to infer unknown parameter values from data, we need a set of high-quality experimental reference data, called the *training data*. The task of parameter identification then becomes an optimization problem: find the parameter values for which the model output reproduces the training data as well as possible. What “as well as possible” means depends on the specific objective. In the ER FRAP example 11, the objective is to minimize the sum of squared differences between experimentally recorded FRAP training curves and the simulation output. Optimization is done using the simplex algorithm 69. The unknown parameter value to be identified is the molecular diffusion constant of the labeled molecules, hence providing a way of inferring diffusion constants in complex geometries (see Fig. 5).

In practice, one often finds multiple parameter sets that work about equally well within the experimental measurement uncertainties. It is then often desirable to find *robust* parameters, i.e. values that would not drastically change if the training data or their measurement errors were slightly different. Quantifying the robustness (or importance) of parameters is the realm of *sensitivity analysis* methods 70. *Local* sensitivity analysis can be used to quantify the robustness of any set of parameter values found by parameter identification. A parameter is called *robust* if it can be varied over a wide range without significantly deteriorating the data fidelity of the simulation output. Because measurement and simulation errors are unavoidable, we usually prefer robust models and robust parameter settings.

If not all of the parameters have been measured or identified, *global sensitivity analysis* 71 can be used to find the most influential parameters in a model, i.e. those that mainly determine the model behavior. This can be useful to figure out which parameters should be measured experimentally. Another use of global sensitivities is to eliminate parameters from a model that have no significant influence on model behavior, hence simplifying the model.

### Verification and validation

Arguably the most important step in modeling is to verify the simulation and to validate the model 72. *Verification* asks the question “am I simulating the model correctly?” whereas *validation* asks “am I simulating the correct model?”

Verification is usually done by considering a simplified system for which the model can be solved exactly (i.e. theoretically). For this simplified *benchmark system*, the simulation result is compared with the theoretical, exact solution and the simulation error hence quantified. No simulation is exact, since we are not solving the model everywhere, but only at the discretization points in space and/or time. However, if one can show that the simulation error decreases with increasing resolution, the simulation is considered verified.

Validation is typically done by comparing with experimental data 72. Of course, the data that are used to validate the model must be independent from the data used to build the model or to identify model parameters. This usually involves performing a set of independent control experiments with perturbations, geometries, or views that were not used for building the model or for identifying the parameters. If the model, with the parameter values identified on the training data, also correctly reproduces these *test data*, it is considered validated. Of course, the validation becomes stronger if more and more different test data are used. No model, however, is correct for all data. It is hence important that both the training and the test data come from within the model's validity frame. It would be wrong to expect a macroscopic model to accurately reproduce microscopic quantities. The continuous ER FRAP model, for example, would never reproduce the trajectory of an individual molecule, as measured in a single-molecule tracking experiment.

The ER FRAP simulations 11 have been verified using a simple 1D benchmark case (see Supplementary Material in 11). Validation has been done by bleaching two different locations in the same ER. The FRAP dynamics from one bleached region was used to identify the diffusion constant of the molecule, and the dynamics from the second bleached region was used as test data to check that the model correctly predicts the influence of the geometric differences between the two regions.

## Conclusions and discussion

I provided an introduction to the terminology, concepts, and challenges of image-based modeling and simulation in biology. The goal is to understand the spatiotemporal dynamics and interactions in biological systems in realistic geometries and shapes. This allows us to directly address questions like: “what is the functional role of shape?” (why, e.g., is the ER a network of tubules and not a spherical blob 9?), “how is shape regulated and generated?” (the question of morphogenesis), or “how do cells organize into tissues, communicate, and arrange?” Several features of biological systems motivate the use of computing. In image-based systems biology the quantitative data used to model shapes and spatiotemporal distributions are extracted from images. This interprets an image as a quantitative measurement rather than a visualization. Once quantitative information is available, it can be used to formulate models of the hypothesized dynamics. The domain of computational models comprises four kingdoms, each with its own capabilities and limitations. Depending on what kingdom a model belongs to, one may have to choose different computational methods to numerically simulate it. Finally, the loop to experiments is closed in model validation and parameter identification.

Many methodological advancements are still required to reach the grand goal of mechanistically simulating an entire cell 73–75 or the development of a complete model organism. The main computational challenges are: (i) flexible image-analysis methods that provide confidence estimates along with the results, that work in more than just one particular case, and that provide a principled way of including prior knowledge about the imaged system and the imaging system. (ii) Multi-scale simulation methods that are easy to use, that correctly couple different scales in the model, and that efficiently utilize modern computer hardware 76. (iii) Robust and efficient black-box optimization and sensitivity analysis methods with proven performance guarantees. (iv) User-friendly software that allows researchers to test different simulation workflows without having to program many lines of code.

Image-based systems biology combines systematic, quantitative imaging with predictive spatiotemporal modeling and simulation. This exposes unique opportunities for developing innovative computational methods, overcoming observability and controllability limits in biology, and understanding biological processes in their natural context.

## Abbreviations

1D: one-dimensional
3D: three-dimensional
ER: endoplasmic reticulum
FRAP: fluorescence recovery after photobleaching
GFP: green fluorescent protein
ROI: region of interest
PSE: particle strength exchange
PSF: point-spread function
